# The thermodynamics of simple biomembrane mimetic systems

**DOI:** 10.4103/0975-7406.76462

**Published:** 2011

**Authors:** Antonio Raudino, Maria Grazia Sarpietro, Martina Pannuzzo

**Affiliations:** University of Catania, Department of Chemistry, Viale A. Doria 6-95125, Catania, Italy

**Keywords:** Biomembrane, DSC, thermodynamics

## Abstract

Insight into the forces governing a system is essential for understanding its behavior and function. Thermodynamic investigations provide a wealth of information that is not, or is hardly, available from other methods. This article reviews thermodynamic approaches and assays to measure collective properties such as heat adsorption / emission and volume variations. These methods can be successfully applied to the study of lipid vesicles (liposomes) and biological membranes. With respect to instrumentation, differential scanning calorimetry, pressure perturbation calorimetry, isothermal titration calorimetry, dilatometry, and acoustic techniques aimed at measuring the isothermal and adiabatic processes, two- and three-dimensional compressibilities are considered. Applications of these techniques to lipid systems include the measurement of different thermodynamic parameters and a detailed characterization of thermotropic, barotropic, and lyotropic phase behavior. The membrane binding and / or partitioning of solutes (proteins, peptides, drugs, surfactants, ions, etc.) can also be quantified and modeled. Many thermodynamic assays are available for studying the effect of proteins and other additives on membranes, characterizing non-ideal mixing, domain formation, bilayer stability, curvature strain, permeability, solubilization, and fusion. Studies of membrane proteins in lipid environments elucidate lipid–protein interactions in membranes. Finally, a plethora of relaxation phenomena toward equilibrium thermodynamic structures can be also investigated. The systems are described in terms of enthalpic and entropic forces, equilibrium constants, heat capacities, partial volume changes, volume and area compressibility, and so on, also shedding light on the stability of the structures and the molecular origin and mechanism of the structural changes.

Impressive progress has been made in detecting and imaging structural properties of biological systems. Structure data, however, represent only the first step toward an understanding of physiological processes. A deeper insight into the functions of biological macromolecules and their supramolecular assemblies requires additional information both on the interactions and on the dynamics governing their behavior. Nowadays, there is renewed interest in addressing the collective behavior of the biological system, shifting the focus from a detailed description of the single isolated molecule to the properties of assemblies of idealized simple objects. Such issues are typically tackled by bio-thermodynamics. At the variance of the classical thermodynamics, where the ultimate goal is the macroscopic properties of a single system (sometimes isotropic and macroscopically homogeneous, such as a liquid solution), biological phenomena involve a variety of multiple scale subsystems, each of them defined over a particular size and time scale. These subsystems, spanning from the angstroms to the micron, and from the pico-second to hours, are not isolated, but strongly interact with each other giving rise to new and challenging phenomena.

In this review we focus on a typical collective system, the biological membrane, selected both for its fundamental role in cell biology and for the different, but closely connected, space and time scales.

In order to be more specific, we list here, a few open questions in membrane science that could be answered only by considering a multiscale approach:

1. Phase diagrams and phase transition kinetics in multicomponent lipid systems — how do we combine observation and modeling of molecular rearrangements on > 100 *nm* length scales during domain formation and / or phase transitions?
2. Coupling between different fluctuating fields (e.g., shape and composition) — how can continuum elastic theories, mean field models, and particle-based simulations be combined so as to capture membrane behavior from 1 *nm* to 10 microns?
3. Cooperative phenomena in membranes — how do membranes and proteins interact collectively in processes that span multiple length and / or time scales, for example, endocytosis?
4. Active lipid transport and non-equilibrium membrane processes in live cells — how is energy efficiently deposited into a membrane to drive processes such as raft domain formation, pore formation, vesicle fusion, membrane invagination, and protein activity?
5. Hydrodynamic effects on membrane dynamics — when are hydrodynamic effects indispensable in membrane dynamics, and how can their effects be quantitatively captured across different scales?
6. Large-scale membrane remodeling events studied through a hierarchy of scales — how do we connect single-molecule diffusion studies to the collective migration of lipid domains or patches?
7. Cross-coupling between lipids and proteins — Membranes move proteins and proteins reshape membranes: how do we systematically improve the minimal protein models and dynamics currently employed in coarse-grained simulations and parametrize them using atomistic modeling?
8. Connecting single / multiple particle tracking experiments with nanoscale spatial resolution in living cells to the underlying collective membrane dynamics. What do such experiments reveal about membrane structure and dynamics?.

The above-mentioned issues are tackled by a combination of theoretical / computational approaches and thermodynamic techniques. On the experimental side, excellent microcalorimeters and other techniques measuring heats, volumes, pressures, and related properties have been developed over the last decades and are now available to a broad spectrum of users.

On the theoretical side, there is an explosion of analytical and computational techniques, which have shown potential usefulness in understanding the collective properties of model membranes. Besides the methods of classical and statistical thermodynamics, new ideas have been proposed, for instance: the theories of phase transitions, the different approaches dealing with out-of-equilibrium thermodynamics, the application of the continuum elasticity, and viscoelasticity theories to lipid membranes and so on. Also on the computational side, a variety of approaches have been suggested in the field of Molecular Dynamics and Dissipative Dynamics. They range from highly idealized coarse-grained pictures of lipids, proteins, and water, to complete simulations at an atomistic level. Simulations are gaining broader and broader applications because they provide, with a steady increasing level of accuracy, information on both the structural details (geometry) and the collective property of the system (e.g., lipid order parameter, total energy, and bilayer elastic constants).

This review is mainly directed to researchers working in the field of lipid membranes in biological as well as model (e.g., vesicle) systems.[1–3] It aims at providing an overview of the thermodynamic techniques and of the physical principles behind the investigated systems. The broad scope of the review makes it impossible to explain the thermodynamic background or technical details of the methods[4–9] or to discuss the results obtained by using them. Instead, the article must be limited to making one aware of the calorimetric assays that are available to tackle a certain problem and to giving a few selected references. One current trend in membrane thermodynamics seems to be the consideration of increasingly complex systems. Vesicles of uncharged DMPC or DPPC (dimyristoyl- and dipalmitoyl-phosphatidylcholine) have yielded important information, but there are many other problems for which these lipids are rather poor model systems. For instance, lipid vesicles made up of ionic lipids and / or pH-modulated vesicle surface potential may represent a useful tool in mimicking the surface potential of real cell membranes. Furthermore, the great interest in lipid rafts has led to a much broader consideration of complex mixtures of glycero-, sphingo-, and glycolipids and sterols. Calorimetry of biological membrane extracts, viruses, organelles or whole cells is being further developed. Another important development is the ongoing introduction of new instruments, techniques, and assays.

The crucial challenge is to combine insights from biochemistry and physiology with those from structural biology and from bio-thermodynamics to derive an integral picture of membranes and their functions. The great amount of experimental data must be interpreted on the basis of approximate, but not over-simplified, models. This issue is too large and cannot be contained in the space of a review. We will mention only the main ideas behind the various thermodynamic models developed to investigate the membrane properties.

## Brief Survey of the Main Thermodynamic Techniques

Calorimeters measure the heat consumed or released by a sample on re-equilibration after a perturbation. Such perturbations can be caused by a change in temperature (Differential Scanning Calorimetry), addition of material (Isothermal Titration Calorimetry), a change in pressure (Pressure Perturbation Calorimetry) or in water activity (sorption calorimetry). For a comparison between different types of calorimeters, such as adiabatic, heat flow, or power compensation instruments, see Wadsö[4] and Höhne *et al*.[10] Briefly, the fast response time of power compensation instruments makes them more sensitive for measuring the heat of fast effects and for revealing their kinetics. Heat flow calorimeters can provide better long-term stability of the temperature and baseline signal, which is particularly important if slow processes are investigated.

### Differential scanning calorimetry

For a detailed introduction to Differential Scanning Calorimetry (DSC), see Leharne *et al*.,[11] although an accurate description of the instrumental apparatus is reported in Privalov *et al*.[12] and Protnikov *et al*.[13] Briefly, DSC records the temperature-dependent isobaric heat capacity, *C_p_(T)*, of a sample. For first order (or weakly first-order) phase transitions, such as the bilayer gel to liquid-crystalline transition, the transition temperature, *T_m_*, is where the heat capacity, Cp, reaches its maximum value. The value of calorimetric enthalpy (∆*H_cal_*) for phase transition is determined by integrating the area under the peak

(1) $$\Delta H_{cal}\;=\;\int \;C_{p}dT$$

From these values, the entropy of the phase transition is determined:

(2) $$\Delta S\;=\;\frac{\Delta H_{cal}}{T_{m}}$$

Comparison of ∆*H_cal_*, ∆*S*, and *T_m_* shows the effect of structural modification (e.g., chain length or ion binding) on the thermodynamics of phase transition. However, unlike a simple organic compound’s crystal to liquid melting transition, the phase transition in bilayers involves more than just the initial and final states. In fact, intermediate ‘states’ are formed during the transition, and a ‘non-two-state’ model is necessary for phospholipids in liposomes.[14–16] These intermediate states result from the formation of domains (e.g., disordered, mobile areas within the gel phase) before the phase transition temperature, and are due to the lateral movement of the phospholipids within the bilayer. The asymmetric shape of the DSC peak reflects the fact that a non-two-state transition has occurred.

In order to adequately fit these data, a ‘non-two-state’ model is required. For any phase transition that occurs between two phases, A and B:

A → B

an equilibrium constant characterizes this process:

(3) $$K=\;\frac{a_{A}}{a_{B}}$$

where *a_A_* and *a_B_* represent the activities (concentration in ideal solutions) of each phase. The temperature dependence of the equilibrium constant is related to the enthalpy by the van’t Hoff equation:

(4) $${\left(\frac{\partial \ln K}{\partial T}\right)}_{P}\;=\;\frac{\Delta H_{vH}}{RT^{2}}$$

The van’t Hoff enthalpy, ∆*H_vH_*, is equal to the amount of heat required for each cooperative unit to undergo the phase transition. The units are energy / cooperative unit. For a first-order two-state transition, the van’t Hoff enthalpy is equal to the calorimetric enthalpy, ∆*H_cal_*. In other words, the heat effect for the transition A → B is the calorimetric enthalpy, which correspondingly governs the distribution between the two phases. If ∆*H_vH_* < ∆*H_cal_* the process involves one or several intermediate stages, such as A → B → C, and is called non-two state. If ∆*H_vH_* > ∆*H_cal_*, the process involves cooperativity, but is not ‘completely cooperative’ as in a first order transition. In other words, the distribution of molecules between the two phases is much more temperature-dependent than the actual heat effect of the phase transition, due to the cooperative motion of the molecules. Therefore, for a non-two-state transition or a partially cooperative transition there are two separate enthalpy parameters, ∆*H_vH_* and ∆*H_cal_*. After subtracting a baseline from the data, which negates any temperature dependence of ∆*H_cal_*, we use equation (4) to obtain an expression to fit our data:[14,16]

(5) $$C_{p}\left(T\right)=\frac{K\left(T\right)\Delta H_{vH}\;\Delta H_{cal}}{{\left(l+K\;\left(T\right)\right)}^{2\;}RT^{2}}$$

where *K(T)* is just the equilibrium constant (3), which is obtained as a function of temperature, by solving (4) for *K(T)*:

(6) $$K\left(T\right)\;=\;\exp\left(-\frac{\Delta H_{vH}}{RT}\left(1-\frac{T}{T_{m}}\right)\right)$$

The software of the DSC apparatus completes this fit and provides the values of ∆*H_cal_*, ∆*H_vH_*, and *T_m_*. For a more physical picture of the van’t Hoff enthalpy, we note that ∆*H_vH_* can be calculated directly from the calorimetric data. First, the Cp versus T output scan from the calorimeter is integrated to form a plot of the enthalpy for the phase transition, ∆*H_cal_*. The maximum of *Cp* versus the *T* curve is *Cp* max. The van’t Hoff enthalpy for the equilibrium is given by: (8)

(7) $$\Delta H_{vH}\;=\;4R{T_{m}}^{2}\frac{C_{pmax}\;\Delta H_{cal}}{\Delta H_{cal}}$$

A sharper transition results in a larger value of ∆*H_vH_*, as *Cp* max is larger. The sharpness of the transition can also be characterized by the full width at half-maximum, of the *Cp* versus *T* peak, ∆T_1/2_. Sharp transitions have a large ∆*H_vH_*, and correspondingly small ∆*T_1/2_*. As the units of ∆*H_vH_* are energy/cooperative unit, and those of ∆*H_cal_* are energy/mole, the ratio of the two (∆*H_vH_* /∆*H_cal_*) gives the value of the moles (or molecules) per cooperative unit:

(8) $$C.U.\;=\;\Delta H_{vH}\;/\;\Delta H_{cal}$$

The larger the value of C.U., the more cooperative the phase transition is. Therefore, cooperative phase transitions have larger ∆*H_vH_*. The value of ∆*T_1/2_* can be used as a qualitative measure of molecular cooperativity. Wider peaks correspond to less cooperative phase transitions. The concept of molecular cooperativity is used for proteins, to determine the number of subunits involved in a transition. The use of this concept for phospholipid bilayers is controversial, but the value of C.U. or ∆*T_1/2_* can give a relative measure of the cooperativity of the bilayer phase transition.

### Isothermal titration calorimetry

The Isothermal Titration Calorimetry (ITC) technique is based on a series of consecutive injections of a liquid sample (a few *µ*l each) from a syringe into the calorimeter cell under isothermal conditions. The heat of the reaction is measured as a function of the injection number, that is, it depends on the concentration of the injectant in the cell. The term ‘reaction’ describes any transition of molecules between different chemical or physical states (including those involving mass transfer inside the solution). Considering that the injection causes ∆*N^Tr^* moles of a compound to undergo a transition accompanied by a molar enthalpy change of ∆*H^Tr^*, therefore, the measured heat q is the sum of the enthalpy changes of all n processes induced by the injection:

(9) $$q-q_{dil}\;=\;\sum_{n}\Delta {N_{n}}^{Tr}\Delta {H_{n}}^{Tr}$$

*q_dil_* denotes the heat of dilution that occurs due to changes in intermolecular interactions of the injectant and of the cell content. These effects are determined by blank runs injecting the titrant into the buffer inside the cell and are eliminated by subtracting the resulting heats.

It is often convenient to work with normalized differential heats, Q, which are given per mole of titrant, ∆*N^Inj^*. In the simple case that only one heat-producing (or adsorbing) process occurs (*n* = 1), we find:

(10) $$Q-Q_{dil}\;=\frac{q-q_{dil}}{\Delta N^{Inj}}\;=\;\frac{\Delta N^{Tr}}{\Delta N^{Inj}}\Delta H^{Tr}\;=\frac{\Delta c^{Tr}}{\Delta c^{Inj}}\Delta H^{Tr}$$

where ∆*C^Tr^* specifies the moles per cell volume that undergo heat-producing transition, and ∆C^Tr^ denotes the change in the concentration of the injectant in the cell caused by the injection. To evaluate the ITC curves, one has to derive a model for the process under investigation that relates ∆*C^Tr^* to the known total concentrations of all compounds in the cell and a few adjustable parameters. Different types of assays can be performed, we refer to the specialized literature for a complete description of these experimental approaches.[8,17–23]

### Pressure perturbation calorimetry

Different calorimeters have been designed for measurement of the heat accompanying an isothermal pressure change, *dQ* / ∂*p*|*_T_*. Such techniques have been referred to, for example, piezothermal analysis,[24] scanning transitiometry,[25,26] pressure jump calorimetry[27] or Pressure Perturbation Calorimetry (PPC).[7,28] A related, adiabatic technique has been termed as volume perturbation calorimetry.[29–32] PPC is mainly used to determine the temperature-dependent, isobaric volume expansion of a sample, *dV / ∂T/_p_*. This approach is based on the Maxwell relation of the reversible heat exchange on a change in pressure, *∂Q_rev_ /∂p* at constant temperature, *T*, to the temperature-induced volume change, *∂V / ∂T*, at constant pressure, *p*:

(11) $$\frac{\partial Q_{rev}}{\partial p}{\left|\frac{}{T}=-T\frac{\partial V}{\partial T}\right|}_{P}$$

Over many years, mainly bulk liquids or solutions were studied on home-built, heat flow calorimeters, mostly using high pressures. Of late, a new generation of PPC instruments have become commercially available as accessories to highly sensitive scanning calorimeters of the power compensation type. The extremely high sensitivity of the calorimeter makes it possible to study changes in the partial volume of as little as ≈1 mg of a protein using only very small pressure jumps of five bars. The first applications of the technique to lipids were studies on the kinetics of phase transitions, on the basis of the relaxation of the temperature or heat changes following a pressure variation (see section Kinetics Phenomena). Volumetric investigations were performed characterizing lipid melting[33–36] and domain formation in membranes.[37]

### Water sorption calorimetry

Different calorimetric techniques have been applied to characterize the enthalpy and free energy of water binding to hygroscopic materials. In all the instruments a lipid film is deposited on the wall of a cell exposed to an atmosphere of varying water vapor activity (the relative humidity, RH). An increase in gas humidity gives rise to an exothermic heat that depends on the molar enthalpy of adsorption from vapor, $∆H_{W}^{vap\to B}$, and the mole number of adsorbed water molecules,

(12) $$q=\Delta {N_{W}}^{vap\to B}\Delta {H_{W}}^{vap\to B}$$

The adsorption of vapor to the membrane is exothermic $∆H_{W}^{vap\to B}\;<0$ and includes: (i) the enthalpy of condensation of water, $∆H_{W}^{liq\to B}\;<0$ = –40.6 kJmol^-1^, (ii) a much smaller enthalpy of binding of liquid water to the bilayer, $∆N_{W}^{vap\to liq}$ :

(13) $$\Delta {H_{W}}^{liq\to B}\;=\Delta {N_{W}}^{vap\to B}\;-\Delta {H_{W}}^{vap\to liq}$$

Thus, from (12) and (13) one can estimate $∆N_{W}^{vap\to liq}$ from independent measurements of *q* and $∆H_{W}^{liq\to B}\;<0$. There exist different techniques to measure the heat *q* and the amount of adsorbed water

### Molar volumes and dilatometry

Direct measurement of the lipid molar volumes and / or their variation with temperature (the thermal expansion α=*V^-1^ dV/∂T_p_* coefficient at constant pressure). This relevant parameter α can be also measured by PPC as discussed in section *Pressure perturbation calorimetry*. Results of molar volume are routinely accurate to the 0.1% level with very good agreement obtained by different researchers using different instrumental approaches. Since the pioneering studies by Nagle *et al*.[44] a consistent number of studies have addressed this relevant topic. Among these studies (often performed by integrating the density measurements with other structural techniques) we mention the still debated problem of lipid-cholesterol mixtures,[45] the nature of the gel to fluid phase transition,[46] the salt effect on the membrane density,[47] and the undulated phase (ripple phase) appearing before the main melting transition,[48,49] the lipid-protein interaction,[50] just to quote a limited number of interesting issues.

### Static and dynamic volume compressibility

In pseudo two-dimensional systems, such as the Langmuir-Blodgett films spread at the water–air interface, lateral compressibility measurements represent the most employed tool used to investigate molecular monolayers. On the contrary, compressibility measurements have been far less used in studying lipid bilayers. Volume compressibility of lipid membranes can be measured by ultrasonic velocity techniques. Briefly, the speed of sound in lipid dispersion depends on the combined compressibility of water and lipid membranes. Thus, in ultrasonic resonators one can calculate the volume compressibility from the wave-length of a standing wave. Consider a membrane being compressed at constant temperature. This means that the heat released on compression is adsorbed and transferred by the surrounding water molecules. For lipid vesicles in an aqueous environment, such a condition is fulfilled if compression is applied very slowly (much slower than the relaxation processes within the membrane), otherwise the measured compressibility is termed as adiabatic compressibility,

$$K\frac{V}{S}.$$

The hydrostatic pressure change, ∆*p*, in the liquid, is proportional to the relative volume change ∆*V / V_o_*

(14) $$\Delta p=K_{v}\left(\frac{\Delta V}{V_{{}^{\circ}}}\right)$$

where *K_v_* is the module of compression. On the other hand, the isothermal compressibility $\kappa_{T}^{V}$ is defined as: $\kappa_{T}^{V}\;=\;V_{o}^{-1}{\left(\partial V/\partial p\right)}_{T}$, thus: $\kappa_{T}^{V}\;=\;1/K_{V}$. The adiabatic compressibility $\kappa_{T}^{V}$ is simply related to the measured sound velocity c by the relationship

(15) $$c\;=\;\sqrt{\frac{l}{\rho {K_{S}}^{V}}}$$

where *ρ* is the sample density. Equation (15) can be easily generalized in the case of a suspension (water + vesicle), enabling one to extract the bilayer compressibility by performing experiments at different water / vesicles ratios. Thermodynamics provide a useful link between isothermal $\kappa_{T}^{V}$ and adiabatic $\kappa_{T}^{V}$ compressibilities

(16) $${K_{S}}^{V}\;=\;{K_{T}}^{V}\;-\frac{T}{VC_{P}}{\left(\frac{\partial V}{\partial T}\right)}_{P}$$

where *C_P_* is the specific heat at constant pressure and *dV* / *∂T*|*_P_* is the isobaric volume expansion of a sample.

A useful property of the compressibility is its relationship to volume fluctuations:

(17) $${K_{T}}^{V}\;=\;\frac{<\;V^{2}\;>\;-\;<\;V\;{>}^{2}}{<\;V\;>\;RT}$$

where < *V* > is the mean volume and < *V^2^* > – < *V >^2^* is the mean standard deviation of the volume. Volume and area (see the next section) fluctuations are very sensitive to bilayer properties, for instance, they increase on decreasing the lipid chain length.[51] However, the most intriguing effect is the divergence of the compressibility at the phase transition. This issue will be discussed a little later. Measurements of isothermal and adiabatic compressibility have been performed on model[52–54] and biological[55] membranes, the results will be discussed later.

### Area compressibility

Strongly anisotropic systems, as lipid bilayers, show a different behavior, depending on whether the force is applied perpendicularly, parallelly or isotropically. Assuming that the energy cost for compression and extension of a membrane about the minimum energy configuration are identical (harmonic approximation), the energy ∆*G* associated with the lateral expansion (compression) area variation is:

(18) $$\Delta G\;=\;\frac{1}{2}K_{A}\;{\left(dA\;/\;A\right)}^{2}$$

where *K_A_* is the area compressibility modulus and *dA / A* is the relative area variation. The force associated with the energy (18) is called membrane tension, τ=*K_A_ (dA/A)*. Direct measurement of *K_A_* is not simple. Nowadays a common method is used, based on the micropipette aspiration technique, developed by Evans and his associates.[56] Typical values of *K_A_* for lipids are in the range of 100 – 200 dyn / cm, but larger values are found in lipid / cholesterol mixtures (for a 1: 1 PC / cholesterol mixture *K_A_ ≈ 800dyn/cm)*. Another elastic constant closely related to the area compressibility modulus described by eq. 18 is the bending elasticity modulus *K_M_*. Indeed, on bending the external leaflet of a lipid, the bilayer expands, while the inner leaflet is compressed and for weak deformations, the contribution of both modes is additive. Theoretical and experimental correlations between the two elastic constants *K_A_* and *K_M_* have been thoroughly investigated (from the standard theory of elasticity: *K_A_* /*K_M_* =*h^-2^*, where *h* is the bilayer thickness).

Analogous to the isothermal volume compressibility discussed in 2.8, the isothermal area compressibility, $\kappa_{T}^{A}\;=\;1/K_{A}$, can also be related to the lateral density fluctuations of a lipid bilayer:

(19) $${K_{T}}^{A}\;=\;\frac{<\;A^{2}\;>\;-\;<\;A\;{>}^{2}}{<\;A\;>\;RT}$$

Such an equation is noticeable and it will be used in section *Passive Membrane permeability*, while discussing the passive transport of lipid membranes.

## Application to Lipid Systems

### Properties of lipid bilayers

Measurable thermodynamic parameters of membranes in their different states (gel, sub-gel, ripple, fluid) are, in particular, the isobaric heat capacity, the thermal volume expansion, and the isothermal or adiabatic compressibilities. It is interesting to compare the thermodynamic properties of lipid membranes with those of the corresponding alkanes of the same length, in order to unravel the peculiar properties induced by bilayer ordering. For instance, absolute heat capacities of different lipid bilayers were determined by Blume[57] using DSC. He found that *Cp* depended strongly on the head group and chain length and the contribution per methylene group in most lipids was larger than in alkanes. The results were discussed in terms of contributions of hydrophobic hydration of the lipid tails to Cp. Furthermore, the thermal volume expansion coefficient of fluid membranes was typically about 10^-3^ K^-1^, a value close, but a little bit larger than that typical to organic solvents. It could be measured with great accuracy by static densitometry in a carefully thermostated heat bath. However, it could also be conveniently measured by PPC as discussed in section *Pressure perturbation calorimetry*. The method determined the volume changes by applying small pressure jumps, which were applied homogeneously to the whole sample. A comparison between the different techniques had been discussed recently.[58]

However, the main difference between isotropic fluids and membranes is that the reduction of the partial volume of the lipid in a bilayer induced by an increase in pressure is highly anisotropic. As more ordered chains can be packed more tightly together, a relatively small reduction in the volume is accompanied by huge lateral area condensation. Consequently, the more ordered straight chains determine an increase in membrane thickness. This means that a typical reduction of the surface area of about 20 – 25%, on going from the fluid to the gel phase[59] is accompanied by a volume decrease as small as 3%. As both bilayer volume (by densitometry or PPC) and thickness (by X-ray or neutron scattering) are available with a great accuracy, the surface area increment is easily calculated. This is an important result, because even subtle variations of the surface area of a lipid bilayer may have a dramatic impact on the morphology of a membrane. Thus, we can define (and measure) three different kinds of compressibilities:

- The volume compressibility (similar to that of the isotropic liquids),
- The area compressibility;
- The thickness compressibility.

Volume compressibility can be easily measured by the techniques described in section *Static and dynamic volume compressibility*. The area compressibility of a bilayer is similar to that measured by lateral pressure measurements in monolayers spread at the water–air interface; there are, however, two main differences: (a) monolayers and bilayers are related, but in different systems; (b) expansion and compression of a lipid bilayer requires comparable energy spending (Hooke law); this is not generally true for it concerns monolayers that monotonously expand against the applied external pressure. For these reasons a direct experimental determination of the lateral compressibility of a lipid bilayer is extremely useful and it can be performed by the techniques described in section *Area compressibility*.

The heat accompanying an area change of the membrane can be measured by ITC experiments injecting vesicles into a hypo- or hyperosmotic solution.[60] The osmotically driven uptake of water into the interior of the vesicles induces an elastic lateral stretching of the membrane, which is endothermic, while the lateral compression of the membrane in a hyperosmotic environment is exothermic.

### Thermotropic phase behavior of pure lipids

Lipid-water mixtures may assume a variety of geometrical structures depending on the nature of the lipids and on the lipid / water content. At high water content the most common structure is the planar lipid bilayer, where, in order to minimize the unfavorable energy associated with water exposure, the bilayer edge assumes an edge-free arrangement: the vesicle. Bilayers can form a large variety of phase structures as a function of chemical composition (including length, branching, and unsaturation of the chains and charge distribution of the heads), temperature, pressure (see below), hydration, and so on. Typical structures at low temperature are bilayers in different subgel, gel, and ripple phases. These phases have stretched acyl chains (i.e., in all-trans conformation) giving rise to wax-like properties. At the main transition or melting temperature, *T_m_*, the ordered phase (L_β_') is transformed into the fluid phase (L_α_). Before the L_β_' ⇔ L_α_ takes place, a phase characterized by undulations of the bilayer surface (the ripple phase) is usually observed, within a narrow range of temperatures. The nature of the reversible L_β_' ⇔ L_α_ transition has been debated over decades. Recent combinations of several experimental techniques, supported by computer simulations, both at the atomistic and coarse-grained levels have shared some light on the detailed mechanism of this complex event that involves several correlated steps, where the final and most important effect is the sharp correlated increase of the entropy-favored gauche conformation of the hydrocarbon tails in respect to the number of ordered trans-conformations. The sharp increase of gauche conformations, however, is not homogeneous along the membrane plane: local patches of melted domains transiently coexisting with solid-like patches appear in the course of the melting event.[61–70] This is the reason for the experimental observation of a divergence of bilayer compressibility values, as discussed in sections *Static and dynamic volume compressibility* and *Area compressibility*.

**Figure 1:**
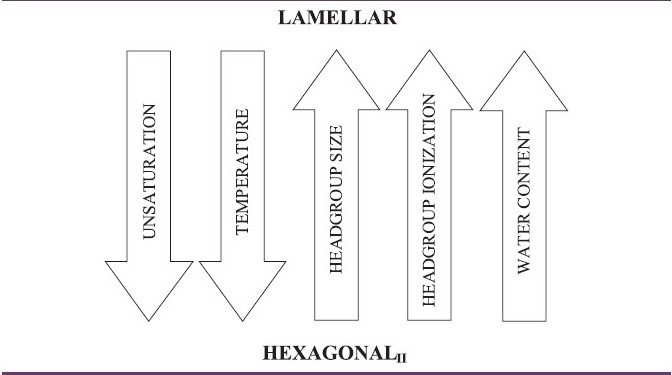
Factors influencing the liquid crystalline bilayer - hexagonal phase preferences of membrane lipids. (Adapted from[71])

Finally, at an even higher temperature, different kinds of lipids form inverse hexagonal phase (H_II_). Sometimes, between the lamellar and the H_II_, lipids form liquid crystalline structures with an astonishing degree of geometrical complexity: the cubic phases. Their structure consists of two mutually interpenetrating, but separate, mesh works of water channels separated by a multiply connected bilayer wall of lipid molecules, organized on a three-dimensionally periodic cubic lattice. The stability of the H_II_ phase depends on several parameters, the main factors influencing the reversible lamellar-to- H_II_ transitions are summarized in Figure 1.[71–73]

Since the pioneering studies of Chapman and others,[74] the standard technique to monitor the phase transitions described above is DSC. Pure lipids usually have very sharp melting transitions with half-widths of the order of 0.05 K. As impurities tend to broaden the transition, the width can be considered as an indicator of purity. Strong membrane curvature in small vesicles as well as undulations or shape fluctuations in large unilamellar vesicles also broaden the transition, and may shift its maximum to (generally) lower temperature. In a similar way, variation in the solvent properties, mainly due to the presence of ions in the solution, may appreciably shift the phase transition temperature (even subtle variations such as the replacement of H_2_O by D_2_O may change the thermotropic behavior,[75]). Over the years, a wealth of lipid melting data has been collected and the effects of chain length, branching, and unsaturation, head group and backbone structure, asymmetry between the two hydrocarbon chains, deuteration or fluorination of the tails, chirality of the lipid molecule, and the like, on *T_m_* and ∆*H*, have been thoroughly studied and modeled. For extensive reviews of phase transitions in different lipid classes, see Koynova and Caffrey’s reviews on glycerolipids,[76] phosphatidylethanolamines,[77] sphingolipids[78] and phosphatidylcholines,[79] phosphatidic acids,[80] and the lipidat data bank.[81] As an example, in Table 1 we report the transition temperature of some lipid bilayers as a function of chain length and unsaturation.

**Table 1:** Transition temperature (in °C) as a function of tail length and saturation. All data are for lipids with the same headgroups and two identical tails[82]

Tail Length	Double Bonds	Transition Temperature
12	0	-1
14	0	23
16	0	41
18	0	55
20	0	66
22	0	75
24	0	80
18	1	1
18	2	-53
18	3	-60

### Thermotropic phase behavior of lipid mixtures

Lipid mixtures can show a very complex thermotropic phase behavior, including eutectic or peritectic points or compound formation.[1,83] DSC is the standard method to establish phase diagrams, by detecting the onset and completion of thermotropic phase transitions. More sophisticated studies have modeled the complete DSC peak, yielding not only transition temperatures, but also thermodynamic non-ideality parameters, describing the interactions (or the associations) in the mixture.[84,85] A very intriguing and biologically relevant system is given by sterols. Molecules such as cholesterol can split the melting transition of phospholipid membranes into a sharp and broad component, suggesting a gradual de-mixing of the membrane. Cholesterol disrupts the lateral order of the gel phase (so), tends to order the liquid phase (ld), and at a higher cholesterol content, stabilizes a new phase, the liquid-ordered phase (lo). This lo phase exhibits both rapid transverse diffusion and translational disorder of the liquid-disordered phase (ld) and relatively orders lipid chains characteristic of the solid ordered phase (so). The overall topology of the obtained phase diagram for binary lipid-cholesterol mixtures has been shown to hold for a range of PC-lipids with both saturated and monounsaturated acyl chains,[86–91] including palmitoyl oleoyl phosphatidyl choline (POPC)-cholesterol mixtures.[88–91] Other sterols as lanosterol and ergosterol have also been found to promote acyl-chain order at high concentrations.[92]

Comparative studies of these three sterols have been conducted and reveal, despite their structural similarities, differences in the effect of cholesterol, lanosterol, and ergosterol on the lipid bilayer properties.[93,94] Similar results have been obtained for other side chain-modified sterols.[95]

Accurate deconvolution procedures give a correct phase diagram of sterols / phospholipids mixtures.[96–99] The properties of mixed bilayers described earlier may also have a deeper impact on other thermodynamic parameters, such as, molar volume and compressibility. For instance, it is known that cholesterol sharply increases the compressibility modulus of phosphatidylcholine bilayers,[100,101] which is accompanied by a rigidification of the chains, as seen by the structural determination of the lipid bilayer thickness.

### Barotropic phase behavior

The fact that lipid phase transitions are accompanied by substantial volume changes implies the existence of pressure-induced phase transitions.[102] Such an effect is the rationale for the well-known adaptation of the lipid membrane composition to extreme pressure conditions observed in deep sea living organisms.[103,104]

In recent times, PPC has become commercially available as another tool to detect lipid melting, which is accompanied by a peak in thermal expansivity. Interestingly, the PPC and DSC peaks of lipid melting exhibit, almost perfectly, the same shape,[33,34,37] suggesting that both the enthalpy and volume of the membrane are governed by the same molecular parameter, most likely the abundance of gauche isomers in the chains. For a more sophisticated discussion of the phenomenon, see Ebel *et al*.[33] The increase in partial volume of the lipid bilayers on chain melting is of the order of 3 – 4%[33,34,105] and the area by about 25%.

Interestingly, many phospholipids with saturated chains of various lengths share the same pressure dependence of the phase transition, *dT_m_* /*dp* ≈ 20 K kbar^-1^, suggesting that this is an intrinsic property of the trans-gauche isomerization of the chains. Hence, this parameter could serve to distinguish chain melting transitions from others. *dT_m_* /*dp* can be determined from a series of DSC scans at various pressures (yielding *T_m_ (p)*), or by comparing ∆*V* and ∆*H* obtained by PPC and DSC according to the Clausius–Clapeyron equation:

(20) $$\frac{dT_{m}}{dp}\;=\;T_{m}\;\frac{\Delta V}{\Delta H}$$

Equation (20) can also be used to compute ∆*V* from the pressure-dependent measurements of *T_m_* and ∆*H*, using DSC.

The sensitivity of a phase transition to pressure can be quantified in terms of the pressure-induced shift of the transition temperature, *dT_m_* /*dp*, or the volume change of the transition, ∆*V.* Both parameters are related to each other according to (20). Shifted transition temperatures of lipids under external pressure have been measured by DSC using pressures ranging from 5 bar to kilobars,[33,34,106,107] yielding *T_m_(p)* and *dT_m_ /dp*. Phase changes of samples have also been induced by pressure jumps at constant temperature (PPC, pressure calorimetry), yielding ∆V of the transition. An increase in pressure can induce a transition from an inverse hexagonal to a fluid lamellar phase (*dT_hex_ /dp* ≈ 40 K kbar^-1^[107]), the freezing of the fluid-lamellar to a ripple phase (≈20 K kbar^-1^ for saturated chains[33,34,106–108] and ≈14 K kbar^-1^ for DOPE[107]), and the pre-transition from the ripple to the lamellar gel phase (≈10-15K kbar^-1^,[33,34,108]).

**Figure 2:**
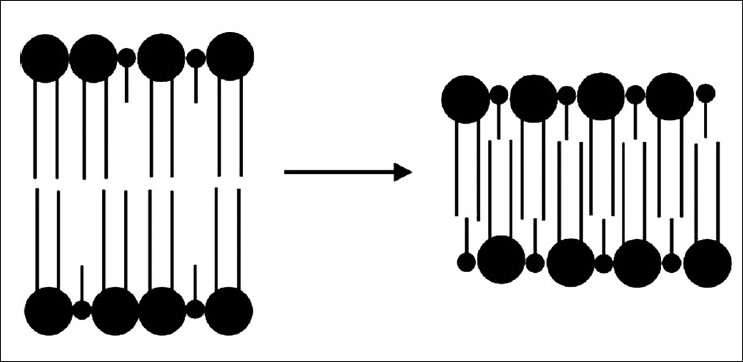
Formation of an intergitaded phase of a two-component lipid bilayer at high pressure

In recent times, Ichimori, Kaneshina, and other authors[109,110] investigated the transition from the pressure-induced transition to the interdigitated phase of phospholipids bilayers [Figure 2].

Figure 2 showing that lipids with two asymmetric hydrocarbon chains or mixtures of long and short lipids easily interdigitate in order to avoid vacancies within the lipid matrix. Finally, an extensive review of the above concepts and experiments but mainly focusing on the interesting issue of the protein behavior at high pressure has been recently reported in the literature.[111]

### Lipid hydration and lyotropic phase behavior

The interactions of the polar and apolar parts of the lipids with water are the driving force for the formation of different phases. Several calorimetric techniques quantify the interaction of water with lipids under different conditions and allow characterizing hydration phenomena in detail.

Water sorption calorimetry determines the enthalpy and entropy of water binding at a given temperature as a function of water activity. It has recently provided valuable insight into the molecular origin of the so-called hydration force, which causes a strong, short-range repulsion between two hydrated (bilayer) surfaces[112–114] due to interfacial water ordering. The ordering of water molecules by lipid–water and water–water interactions, as well as the entropy gains arising from fluctuations in the membrane structure, have been discussed as the basis of hydration forces. For DOPC bilayers, sorption calorimetry showed that only one or two water molecules per lipid exhibit an exothermic binding at 25°C, that is, these are bound and ordered by specific interactions. The adsorption of the remaining water molecules onto the lipid molecules is endothermic and is therefore driven exclusively by an entropy gain. Hence, water bound to the lipid increases its motional and conformational freedom, and the resulting entropy gains must also be considered on the basis of the hydration force. This important conclusion is further supported by the sorption calorimetric studies of POPC[38] and a series of saturated lipids showing three to four enthalpically bound water molecules per lipid.[40]

The thermodynamics of a lyotropic gel-to-liquid crystalline transition of POPC at low relative humidity have also been discussed on the basis of sorption calorimetry.[38] The enthalpy change accompanying a lyotropic lamellar-to-hexagonal transition depends on whether the lipid forms direct hydrogen bonds or not.[115]

Another approach for determining the hydration pressure of lipid phases is to record the phase transition temperatures at different, well-defined, water contents, by DSC (see, for instance the pioneering work by Cevc and Marsh[116]). For more recent studies on this issue see the study by Pfeiffer *et al*.[117] By calculating the mean pressure among the planar neutral bilayers brought close to each other on account of water ordering at the membrane surface, they found that the shift in temperature of the gel-to-liquid crystalline transition of lipid membranes behaves as

(21) $$\Delta {T_{t}}^{hyd}\;=\;\Delta {T_{t\infty }}^{hyd}\;\tanh\left(\frac{n_{w}V_{w}}{\xi S_{L}}\right)$$

where *n_w_* is the number of bound water molecules, *V_w_* the volume of one water molecule, ξ the correlation length of water polarization (a measure of the decay of water orientation on going from the membrane surface to the bulk phase), *S_L_* the lipid area, and the $∆T_{t\infty }^{hyd}$ transition temperature shift at limiting hydration (nw → ∞), which is independent of water content. tanh(χ) is the hyperbolic tangent function (tanh → (X) as x<<1 and tanh(χ) → 1 when χ → ∞). These theoretical results agree well with the DSC measurements performed at a controlled water content.[116]

Finally, a characteristic number of lipid-bound water molecules, called ‘unfreezable water’, can be deduced from the enthalpy of water freezing / melting of a sample of well-defined water content.[118–120]

### Self-asso ciation of lipids

The critical micelle concentration (c.m.c.) and enthalpy of micelle formation, ∆*H_mic_*, can be determined by titration calorimetric experiments. From measurements at varying temperatures, the heat capacity change, ∆C_p,mic_, is also derived. Unfortunately, such an approach has limited application in the investigating lipid vesicles, because typical membrane lipids have critical association concentrations in a range that is not accessible by the ITC. Studies have, however, been performed on shorter chain analogs like diacylphosphatidylcholines and lysophosphatidylcholines.[121] The results have been discussed in terms of group contributions to enthalpy and free energy of self-association and changes in the water-accessible surface area of lipids. Furthermore, they have shown that the alignment of the acyl chains in an aggregate gives rise to a significant change in enthalpy (but not in free energy) compared to the state in bulk hydrocarbon. This finding is also important for the interpretation of enthalpies of insertion of molecules into lipid membranes.

## Membrane Partitioning and Binding of Additives

### Modification of the membrane phase diagram by solutes

Small molecules, drugs, peptides, and proteins are not in general readily soluble in the solid-like phase of the lipid bilayer due to their crystalline structure. They are much more soluble in the fluid-like phase. This leads to the well-known reduction of melting points, demonstrated in the early seventies by a number of authors.[122] This effect is known as the vant’Hoff freezing point depression. For example, the solubility of NaCl is high in water and low in ice. Thus, salt lowers the freezing point of water. This effect is due to the difference in mixing entropy of the ions in water and ice. For low solute concentrations and with reasonable assumptions of perfect miscibility of lipids and solute in the fluid-like phase and immiscibility in the solid-like phase, one arrives by classical thermodynamics at the well-known relation between melting point depression and solute concentration

(22) $$\Delta T_{m}\;=\left(\frac{R{T_{m}}^{2}}{\Delta H}\right)\log\;a_{w}\;\approx \;-\left(\frac{R{T_{m}}^{2}}{\Delta H}\right)X$$

where ∆*H* is the lipid-melting enthalpy of the lipid bilayer (about 35 kJ/mol for DPPC), *R* the universal gas constant, and *T_m_* the lipid-melting temperature (314.3 K for DPPC), a_w_ is the solvent activity related to the molar fraction of the impurity inside the membrane X by the relationship: logaw=log(*γ_w_(1-X)*). In the simplest approximation, the water activity coefficient log *γ_w_* =0, but other choices can improve the analysis (see in the following text). Several DSC data can be interpreted with the aid of eq. (22). There are, however, several points to consider:

- The solute is soluble both in the gel and fluid phase, the greater solubility occurring in the fluid phase. When the solute has the same solubility both in solid-like and fluid phase, then ∆*T_m_* =0.
- There is a solute partitioning between the water and the membrane (see the next section). The net effect is a decrease of the solute concentration, which now depends on the lipid / water ratio.
- Solute-membrane mixing is not ideal (see section Non-ideal mixing). In this case the solvent activity coefficient is no longer zero and it does depend on solute concentration: log*γ_w_≈ A=CX^2^* +.... The shift of the melting temperature, ∆*T_m_*, with the solute concentration *X* assumes a typical parabolic shape, often observed in DSC experiments.[123]
- There are no structural variations in the bilayer structure. Solutes, for instance, may induce interdigitation among the tails of the lipid leaflets[124] or other morphological phase transitions toward non-planar shapes.

Despite these serious limitations, the vant’Hoff-based picture of the depression of the freezing point can be useful in studying the solute-induced variations of the membrane transition temperature, because it is conceptually simple and straightforward for practical purposes. Improvement of eq. (22) can be reached either by remaining in the realm of classical thermodynamics (for instance, by introducing a partition coefficient for the impurity between the melted and un-melted lipid phases as done by Inoue[125] or by introducing a phenomenological non-ideal mixing enthalpic term[123] and discussed by us later). Alternatively, one can shift toward a microscopic modeling of the lipid-impurity interactions as pioneered by Mouritsen *et al*.[126] The physics behind the solute-induced temperature shift of phase transitions is common also to other systems. For instance, statistical mechanical theories, similar to those employed to explain the shift of the main transition in lipid bilayers, were developed by Crothers and McGhee. They allow a simple interpretation and calculation of DNA melting curves (detected by DSC techniques) in the presence of ligands or proteins.[127–129]

### The partitioning of non-ionic solutes into membranes

Several techniques (e.g., radio-labeling or spectroscopic techniques) can be employed to investigate a solute binding to a membrane. Among them, ITC has become a standard method for characterizing ligand binding.[130] For this assay, a solution of a compound A filled into the cell is titrated with the solution of a different compound B loaded into the syringe. By making use of a proper model equation it is easy to fit the data obtaining the binding constant, *K_o_*, the molar enthalpy change, ∆*H*, and the stoichiometry of the reaction. This model is appropriate for binding the ligands to the receptors residing in the membrane. Similarly, solute partitioning into membranes can be studied very favorably by different types of ITC assays. The process giving rise to the heat, *Q*, is the transfer of solute (S) molecules from the water (w) to the lipid bilayer (L), which is accompanied by a molar enthalpy difference, $∆H_{S}^{w\to L}$. In a similar manner, one can measure the release of a solute from the bilayer, accompanied by an enthalpy change $∆H_{S}^{L\to w}\;=\;-∆H_{S}^{w\to L}$. Hence, the transferred concentration ∆*c^Tr^* in equation (10) has to be replaced by the change in concentration of the bilayer-bound solute, ∆*C_S_*, derived on the basis of a lipid / water partition coefficient *K_o_*. A variety of definitions have been used for the partition coefficient; for a detailed discussion.[131]

A good description of the partitioning of amphiphiles is often possible in terms of a constant mole ratio partition coefficient, K_o_, obtainable by standard thermodynamic arguments:

(23) $$K_{{}^{\circ}}\;=\frac{C_{S}}{C_{L}{\overset{-}{C}}_{S}}=\frac{C_{S}}{C_{L}\left(\;{\overset{-}{C}}_{S}\;-C_{S}\right)}$$

where the symbols *C_S_* and *C_L_* denote the molar concentrations of solute dissolved in the lipid bilayer and that of lipids, respectively, (virtually, lipids are completely located in the bilayer because of the extremely small CMC of most lipids). ${\overset{-}{C}}_{S}$ denotes the molar concentration of the solute dissolved in water and is related to its stoichiometric concentration.

Most ITC partitioning assays are based on injections of lipid vesicle suspensions into the calorimeter cell. For the uptake protocol,[132–135] the cell contains the buffer-dissolved solute, so that every aliquot of lipid vesicles injected into the cell binds a fraction of the remaining free solute. The release protocol[136,137] is based on small injections of lipid vesicles containing solute into a large excess volume of buffer; the dilution gives rise to a release of solute from the bilayers. A model equation that allows one to fit the uptake data has been derived, resulting in

(24) $$Q=K_{{}^{\circ}}\frac{C_{S}}{{\left(l+KC_{L}\right)}^{2}}\Delta {H_{S}}^{W\to L}\;+Q_{dil}$$

Similar equations can be obtained for what concerns the release protocol. The above model assumes that *K_o_* and $∆H_{S}^{w\to L}$ are independent of solute and lipid concentration. In many cases, the solute mixes non-ideally with the lipid (see section Non-ideal mixing), therefore these assumptions are not a priori warranted, more refined models allowing for composition-dependent *K_o_* and $∆H_{S}^{w\to L}$ have been used,[138–140] but in most cases the experimental data do not justify the introduction of other adjustable parameters (such as a non-ideality parameter). However, it must be noted that a two-parameter model, equation (24), yields good data even if the model assumptions are not strictly fulfilled. For a more detailed discussion and partitioning data for many systems, see articles on membrane binding of peptides,[133,141] surfactants,[131,142,143] alcohols,[144–147] and drugs.[148,149]

The knowledge of the partition coefficient enables one to calculate the apparent standard chemical potential change of a solute, on transfer from water into the lipid bilayer, $∆{µ}_{S}^{0,w\to L}$, which is obtained as

(25) $$\Delta {{µ}_{S}}^{0,\;w\to L}\;=-RT\log\left(K_{{}^{\circ}\;}C_{W}\right)$$

with the water concentration in dilute solutions, *C_W_* = 55.5 M.[131] The contribution to $∆{µ}_{S}^{0,w\to L}$ that arises from the hydrophobic groups that are buried in the apolar core of the membrane is similar to that obtained on self-association with micelles (≈3 kJ mol^-1^ per methylene group). It is worth mentioning that the enthalpy and heat capacity changes on membrane insertion are quite different from those of micelle formation, indicating that changes in lipid packing caused by the solute may have substantial consequences.

Application of these studies is the understanding of well-known, but still elusive issues. One of them is anesthesia, a phenomenon caused by a number of small molecules that partition in the biological membrane. For more than a hundred years it is known that the effectiveness of anesthetics is proportional to their solubility in olive oil (that has the properties of the membrane interior). This rule is known as the Meyer-Overton rule.[150] It holds over several orders of magnitude ranging from laughing gas, N_2_ O, over halothane to lidocaine. Even the noble gas xenon is an anesthetic. This observation excludes any specific binding to macromolecules (e.g., proteins) if one is searching for a generic explanation of anesthesia. It has also been known for a long time that anesthetics cause a lowering of phase transition temperatures.[151,152] There are strong indications that the effect of anesthetics is related to this finding. As shown in section *Barotropic phase behavior*, phase transitions are pressure-dependent. Even as pressure increases transition temperatures, anesthetics lower them. It has in fact been found that pressure reverses the effect of anesthesia.[153]

### Membrane binding of small or large charged solutes

For charged solutes one has to take into account that the aqueous concentration of the solute is in the vicinity of the membrane, which is in equilibrium with the membrane-bound solute, and differs from that in the bulk solution, ${\overset{-}{C}}_{S}$. The apparent partition coefficient, *K_app_*, strongly depends on the electrostatic potential of the membrane surface with respect to the bulk, *ψ_o_*, and the charge number of the solute, *Z_S_*:

(26) $$K_{app}\;=\frac{C_{S}}{C_{L}{\overset{-}{C}}_{S}\;}=K_{{}^{\circ}}\;\exp\left(-\frac{z_{S}e\psi_{{}^{\circ}}}{K_{B}T}\right)$$

with e and k_B_ denoting the elementary charge and the Boltzmann constant, respectively. The potential ψ_o_ depends, in turn, on the ionic strength and the bound solute. It can be determined on the basis of the Gouy-Chapman theory, which relates *ψ_o_* to the solution ionic strength, dielectric permittivity e of the solvent, and electrical surface density σ (number of charged lipids / lipids area), through a relationship derived from the electroneutrality condition of the whole system: *ε(∂ψo/∂z)* = σ (the derivative being performed with respect to the z-axis perpendicular to the surface). In case of the weak surface potential, the above charge density-potential relationship yields: *ψO* = *σ/εκ*, where *κ = (2e^2^ c/(ek_B_T)^1/2^* is the Debye constant, proportional to the salt concentration, c; more general complex relationships between σ and *ψ_o_* valid at high potentials can be derived as well. If the intrinsic partition coefficient, *K_o_*, which does not depend on electrostatics is known,[133,154,155] measurements of K_app_ can give information on ψ_o_ (or σ) and vice versa. The thermodynamics of ionization of a lipid on NaOH addition[156] and the ion adsorption to lipid bilayers[157] were studied by ITC. A variation in the buffer used in ITC partitioning or binding experiments can be used to reveal protonation–deprotonation effects accompanying ligand binding to membranes. This approach is based on the fact that the protons released or bound by the ligand are absorbed or provided by the buffer, respectively, so that the heat of ionization of the buffer contributes to the measured heat of titration. As the protonation heats of many buffers are known,[158] the apparent heat of binding in different buffers can be plotted versus the heat of buffer protonation, yielding the change in protonation and the intrinsic heat of binding.[133,154,159–163] It should be noted that the assumption of a constant average membrane surface potential (the Gouy–Chapman Theory) is an approximation leading to good results in most cases. Nevertheless, the local potential may be different, in particular for ligands that carry many charges. However, even for single-charged ligands the assumption of a constant potential fails at high surface coverages. As the surface potential ψ_o_ is proportional to the effective surface charge density *σ : σ = σ_o_* (1-ZC_S_), with σ_o_ the charge density at zero coverage, Z the ligand charge, and *C_S_* its surface concentration. We conclude that the effective binding constant defined by eq. (26) does depend on concentration C_S_. This effect is stronger on increasing the ligand’s net charge Z. Charged ligands such as polyelectrolytes or peripheral proteins exposing many positive charges toward the membrane surface may accumulate negatively charged lipids in a mixed membrane of anionic and zwitterionic lipids. Such effects have, for instance, been discussed in detail on the basis of ITC data on cytochrome C[164] and annexin/Ca^2+^.[165] Further indirect evidences for the accumulation of charged lipids in mixed membranes are based on DSC measurements. Some examples were described by the authors.[166–168] In a similar manner, the binding of DNA to membranes containing cationic lipids has been characterized by ITC, revealing the thermodynamic parameters of the entropy-driven interaction as well as critical charge ratios[169] and protonation effects.[163] The effect of the interaction is also evident on the DSC curves that are shifted and split on DNA addition.[169] For a recent comprehensive thermodynamic analysis of macroions ‘decorated’ lipid bilayers see the study of May.[170]

## The Effects of Additives on Membrane Properties

### Non-ideal mixing

The free energy of mixing in fluid membranes is often close to the ideal value, as enthalpic and entropic interactions balance each other to a considerable extent (see next section on phase separation). The enthalpy of mixing is, therefore, a much more sensitive parameter for investigating the non-ideal mixing behavior of membrane constituents. We may write the mixing enthalpy *h* of a two-component system, A and B, with X denoting the mole fraction of B, as

(27) $$h\;=\;N_{A}H_{A}\;\left(0\right)\;+N_{B}H_{B}\left(1\right)+\left(N_{A}+N_{B}\right)H_{EXC}\;\left(x\right)$$

*H_B_* (1) and *H_A_* (0) stand for the molar enthalpies of B and A in pure systems. In the simplest approximation

(28) $$H_{EXC}\;\left(x\right)=\frac{1}{2}Z\left(w_{AA}X^{2}\;+\;2w_{AB}X\left(1-X\right)+w_{BB}{\left(1-X\right)}^{2}\right)$$

where *z* is the number of contacts around each molecule and *w_ij_* represents the pair interaction energy between the i-th and j-th molecules. For ideal mixing, *h* is just a linear combination of the enthalpies of the lipid and solute, and the excess enthalpy, *H_EXC_(X)*, vanishes. Non-ideal mixing is represented either by *H_EXC_(X)<0* if the A–B contacts are enthalpically favorable, or by *H_EXC_(X)>0* if A–B mixing is enthalpically unfavorable. If the pure components are in only one state (we neglect, for instance, the partitioning effects or the occurrence of micellization), *H_B_* (1) and *H_A_* (0) are independent of the absolute concentration. The normalized heat, *Q*, of the injection of a pure component (A or B) into the mixture was derived in Heerklotz *et al*.[171], yielding

(29) $$Q=\left(1-X\right)\frac{dH_{EXC}\left(X\right)}{dX}+H_{EXC}\;\left(x\right)$$

with *X* denoting the mole fraction of the injectant (B) in the mixture. Hence, the heats measured on titration of the solute into the lipid and those measured on titration of the lipid into the solute can be used to derive the same excess enthalpy function, *H_EXC_(X)*, by solving equation (29). This was done for a series of lipid–detergent systems.[171] As one might expect, bilayer-forming additives show small non-ideality effects in lipid bilayers, but micelle-forming solutes mix highly and non-ideally with lipids in membranes, *H_EXC_(X)*> 0.

It is important to stress the difference between the state of the system (characterized by *H_EXC_(X)*) and the heat *Q* representing the partial molar enthalpy. Positive values of *Q* do not necessarily mean that the mixing is unfavorable, but only that the addition of a compound renders the enthalpy of mixing less favorable.

The enthalpy of mixing in a membrane can also be studied through a detailed analysis of the shape of the DSC curves of lipid transitions. Studies of lipid mixtures have been discussed in section *Lipid mixtures*. The thermodynamic analysis of the melting point depression with the concentration of an additive inside the membrane developed in section *Modification of the membrane phase diagram by solutes* can be extended to the case of non ideal lipid-additive mixing. A simple thermodynamic calculation gives the leading terms:

(30) $$\Delta T_{m}=-\left(\frac{R{T_{m}}^{2}}{\Delta }\right)X\left(l-wX\right)$$

where *w ≡ z(w_AA_* +*w_B_* - *2w_AB_*) is the non-ideal mixing parameter, while the other terms have been defined in eq. (22).

The physical origin of non-ideal mixing is strictly related to the molecular structure of the binary bilayer single components. Differences in the hydrocarbon chain length, nature, and charge of the head groups and ion adsorption by a specific lipid component[172] may dramatically change the mixing behavior. Similar investigations have also been performed for lipid–protein membranes containing, for example, bacteriorhodopsin,[173] cytochrome C,[164,174] gramicidin A,[175] glycophorin,[176] and tetanus toxin.[177] Some simple cases of peptide mixtures with lipid membranes were discussed by Ivanova *et al*.[27,178] For a recent review of the DSC data on lipid-protein mixtures see, e.g., Lewis and McElhaney.[179]

As a rule of the thumb, it can be shown that the influence of peptides or proteins on the specific heat profiles is mainly due to the miscibility of the peptide with the two lipid phases, gel and fluid, respectively. If, for instance, the peptide mixes well with the fluid phase (low nearest neighbor interaction energy) and does not mix well with the gel phase (high nearest neighbor interaction energy), the peptide will homogeneously distribute in the fluid phase, but aggregate in the gel state. The corresponding heat capacity profile will be shifted to lower temperatures and display an asymmetric broadening at the low temperature side of the transition.[27]

### Lateral phase separation: Different routes to domains formation

The problem of whether molecules mix randomly or tend to form clusters of certain compositions or arrangements is governed by the excess free energy defined as the difference between the energy of the mixed state and that of the pure components: *G_EXC_* = *H_EXC_ - T∆*S*_MIX_*. *H_EXC_* has been defined by eq. (28), while the mixing entropy ∆*S_MIX_(X)* reads: ∆*S_MIX_(X) = -k(N_A_* log *N_A_* + *N_B_* log *N_B_*) = *kN (X* log *X* + (1-*X*) log (1-*X*)), with *k* being the Boltzmann constant and *N* the total number of molecules. Many lipid-additive systems showing non-ideal enthalpies of mixing can nevertheless be well described as randomly arranged mixtures, as the endothermic enthalpies of interaction are essentially balanced by the gains in mixing entropy. The fact that many additives exhibit a virtually constant mole ratio partition coefficient into lipid bilayers,[133] implies slightly unfavorable excess free energies of *G_EXC_* ≤ 0.4 kJmol^-1^. However, this non-ideality does not give rise to significant deviations from random mixing, because *G_EXC_* is small compared to the thermal energy (≈2.5 kJmol^-1^ at room temperature).

**Figure 3:**
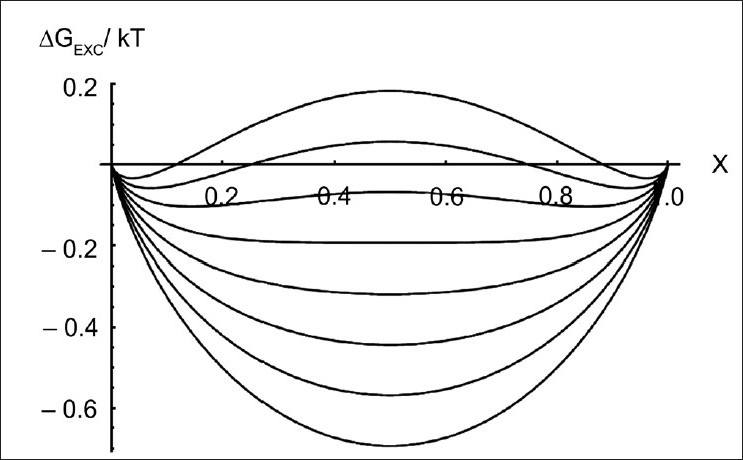
Variation of the excess free energy against the composition *X* of a fluid binary mixture. The curves have been calculated for increasing values of the non-ideal mixing parameter *w/kT*. The first curve on the bottom corresponds to w/kT = 0

Combining the explicit expression for the mixing enthalpy *h*, eqs.(27,28), with that of the mixing entropy reported earlier, we calculate the excess free energy as a function of the mixture composition *X*, its plot is given in Figure 3.

**Figure 4:**
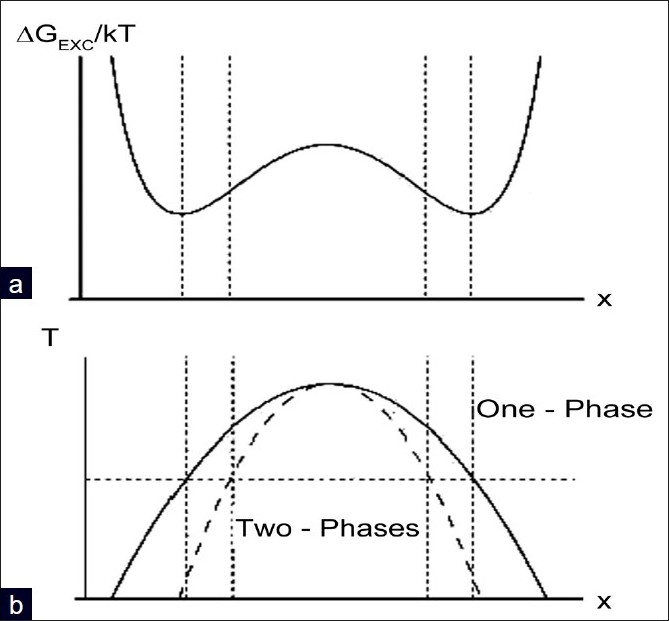
Panel A : variation of the temperature scaled excess free energy against the composition X of a binary fluid mixture. Panel B : Phase diagram of a binary fluid. The continous curve is the locus of the minima of panel A (binodal curve, ∂G_EXC_(X)/∂X=0), while the dashed curve is the locus of the inflexion points (spinodal curve, ∂G_EXC_(X)/∂X^2^=0). To is the composition-dependent temperature at which phase separation takes place

It can be easily seen that, depending on a single parameter *w/kT* ≡ *z(w_A_* +*w_B_–2w_AB_)/kT*, either a single minimum or two minima separated by a maximum can be observed. The position of the minima (and maxima) is calculated by imposing: ∂*G_EXC_(X)/∂X*=0, from which we get:

(31) $$\log\frac{X}{l-X}=\frac{w}{kT}\left(l-2X\right)$$

(extension to mixtures of molecules differing in size is straightforward). Their locus as a function of *T* define the equilibrium curve (binodal curve, full line), which separates the one-phase and two-phase regions as reported in Figure 4, panel B.

Furthermore, unstable regions of negative curvature (∂*G_EXC_(X)/∂X^2^* <0) lie within the inflection points of the curve ∂*G_EXC_(X)/∂X^2^* =0, which are called the spinodes. Their locus as a function of temperature defines the spinodal curve reported in Figure 4b (dashed line). As we shall see shortly, the difference between the binodal and spinodal curve has a key influence on the final morphology of the phase separated system.

Although thermodynamics fixes the conditions for phase separation to occur in a rather transparent manner, the mechanisms leading to phase separated samples and their final morphology require a combination of purely energetic considerations, together with a dynamic picture of the whole process.

The two most common pathways to phase separation are nucleation mechanisms or spinodal decomposition mechanisms. Starting from a point inside the one-phase region, a change in any physical parameter (e.g., temperature) brings the system inside the two-phase region of phase in Figure 4. In the region, enclosed between the binodal and spinodal curves, phase separation occurs through the nucleation and growth mechanism, a process controlled by the undercooling temperature. As discussed later, nucleation is a slow mechanism because it requires to overcome an undercooling-modulated energy barrier. A deeper cooling beyond the spinodal curve, brings the fluid to a phase separated structure through a mechanism that involves the increase of the composition fluctuations inside the binary fluid. The final morphology associated with the two processes is different:

- The nucleation mechanism leads to spheroidal isolated droplets richer in one component.
- Spinodal decomposition leads to inter-connected domains of different compositions.

These different topological patterns may bring to noticeable different in the membrane structure and function. Consider, for instance, the lateral diffusion of a tracer in patterned A-rich and B-rich membrane domains and assume for simplicity that diffusion takes place only in the B-rich domains. If the B-rich domains are disconnected (i.e., they come from a nucleation and growth mechanism) the diffusant remains trapped inside these micro-pools and cannot reach the target. On the contrary, in a connected structure of A-rich and B-rich microdomains the diffusion is slow, but finite: the diffusant may reach the target.

Domains formed by spontaneous de-mixing of lipids in a membrane have recently become a focus of interest, as such domains in biological membranes, referred to as ‘lipid rafts’, are believed to have important biological functions.[180–183]

Calorimetric techniques, and in particular DSC, provides a useful tool to detect the formation of laterally heterogeneous structures within the lipid membrane. Consider a two-component lipid membrane that contains micro-domains richer in one component. If the domains are large and stable on the time scale of the main lipid transition, the response to constant heating (or cooling) will be markedly different: the melting temperature of each domain will be similar to that of the more abundant component. This fact leads to a broadening or even the splitting of the calorimetric peak, which is proportional to the composition of the domain and to the properties of the separated lipid components (temperature transitions and enthalpy of pure components).

By exploiting the above -mentioned ideas, a large variety of phase-separating lipid systems have been investigated by DSC. Lipid partial immiscibility may arise from the different interactions occurring among the head groups, due to electrostatic interactions, or from different interactions among the tail due to their different length or unsaturation along the hydrocarbon chains. This effect is easily understandable and it is conceptually similar to that found in binary poorly mixable liquids. However, when mixed lipid bilayers and lipid / protein membranes are considered, a new strong, indirect force, favoring micro-domain formation, emerges. The physical basis of these forces, sometimes termed as hydrophobic mismatch, has been introduced by Mouritsen and his co-workers.[184] Several authors have confirmed and further improved the original theory of Mouritsen.[185–192]

It has been assumed that these rafts can be isolated from the membranes by detergents. ITC studies of the enthalpy and entropy of interaction of the detergent *triton* with different lipids imply, however, that the addition of a *triton* to the membrane, changes the degree of domain formation and the composition of the domains substantially.[193] The predicted exothermic process of *triton*-induced formation or growth of domains could indeed be detected by ITC, and the stabilizing effect of the triton on these domains can also be measured by DSC and PPC.[193]

Some years ago, Melchior[194] proposed a useful calorimetric trick to investigate inhomogeneous lipid membranes. The application of rapid-freezing techniques to DSC provides a new approach for understanding the organization of lipids in biomembranes. Use of quick-freeze DSC on membranes of mixed lipid composition supports the existence of nonrandom distributions of lipids (domains) in fluid bilayers. In addition to allowing investigations on the organization of lipids in the fluid bilayers, the quick-freeze technique now allows calorimetric studies to be carried out on mammalian membranes, which, because of their high cholesterol content, have not been previously amenable to the study, by DSC.

Differential calorimetry experiments provide extremely useful thermodynamical parameters to characterize the temperature behavior of lipid mixtures. However, as the number of components in the lipid mixture increases, data analysis becomes very difficult. Although thermodynamic information from the DSC experiment can be extracted from the system, no detailed information about the physical characteristics of lipid lateral structure at different temperatures can be obtained using this technique. Nowadays, fluorescent techniques and scanning microscopies provide additional information not available by thermodynamic measurements.

### Coupling between lipid domains and membrane properties

An astonishingly large number of membrane properties are modulated by the formation of lipid domains.

One of the most challenging topics involves the possibility that local concentrations of lipids having different shapes could couple with membrane curvature to produce a sorting mechanism of the different membrane components. This issue presents us with the following questions: How strongly curved must a membrane be to produce a sizeable sorting effect? The counterpart of this question is: How does the effect of this curvature vary with the degree of asymmetry of the lipid composition? Among the rapidly growing literature in this relevant field, we have just quoted some of the more recent articles.[195–197] The lateral phase separation-bilayer shape coupling plays a key role in explaining a variety of biological relevant phenomena such as hexo- and endocytosis, for space reasons we will not analyze such a broad field.

### Effect of curvature strain on the thermodynamic properties of membranes

Membrane curvature effects of inclusion compounds have been found to play an important role in biological membrane function,[198–202] and therefore, they have been widely explored over the past decades. It has turned out that most of the membrane-ordering or disordering effects of additives can be interpreted in terms of a relaxation or induction of curvature strain. The general background of these phenomena can most easily be illustrated by Israelachvili’s concept of ‘effective molecular shapes’ dictated by the ratio of the lipid surface area / maximum stretching length (under the hypothesis of substantial volume incompressibility of the hydrocarbon chains that are assumed to adopt a liquid-like arrangement in all the geometrical conformations of the lipid aggregate).[203] It is worth recalling that the maximum stretching length is a property of a single lipid molecule, but the lipid surface area strongly depends on the lateral collective interactions of the lipid heads and on the solvent properties. Molecules such as POPC pack together to a planar arrangement, as the surface area required by two fluid chains (≈ 2 × 27 Å^2^) agrees fairly well with the surface area occupied by the PC head group (≈ 61 - 65 Å^2^). Surfactants with a large head group, but only one acyl chain are referred to as ‘inverted cone-shaped’; they pack together to form a strong, positively curved (convex), micellar surface. Molecules such as DOPE, with a small head group and a hydrophobic part requiring a relatively large surface area, tend to form curved surfaces with the hydrated heads in the center; these are called inverse or negatively curved structures. Although the preferred, ‘spontaneous’ curvature varies gradually, the choice of surface geometries that can be realized by stable aggregates is limited. The average real curvature of a lipid bilayer of a large vesicle is practically zero, but that of other (e.g., micellar or cubic phases) geometries differs substantially from zero. The difference between the spontaneous curvature of the constituents and the real curvature of the resulting aggregate is called a ‘curvature strain’. As a rule, enthalpies of membrane insertion of additives measured by ITC have been found to be more endothermic if the curvature strain they create in a membrane is more.[204,205]

Similar results have been seen when comparing the DSC data of phospholipids bilayers with different radii. Experimentally, depression and broadening of the phase transition temperature is observed for strongly curved vesicles. For vesicles smaller than ~70 nm in diameter the phase transition temperature gradually decreases with decreasing vesicle size.[204,206] Similar effects have been detected by using densitometric techniques[207] or for bilayers deposited into nanopores.[208]

Additives that can relax a pre-existing curvature strain may bind exothermally.[204] These results suggest that the excess enthalpy, H_EXC_, of a bilayer (see section *Non-ideal mixing*) is governed by the curvature strain.

Although non-ideal mixing and general membrane ordering are strongly related to the curvature strain, a more specific interpretation of spontaneous curvature effects is possible, considering the lamellar-to-inverse hexagonal transition of suitable model lipids (e.g., POPE), as the latter is accompanied by a real change in curvature from zero (lamellar) to negative values (inverse hexagonal). Compounds that induce positive spontaneous curvature favor the lamellar phase and increase the transition temperature, *T_hex_*, whereas, substances inducing negative spontaneous curvature promote the curved phase and decrease *T_hex_*.[209–212] Recent applications of these concepts have been proven to be useful in investigating cationic membrane-DNA complexes.[213]

### Passive membrane permeability

A biological relevant phenomenon is the passive permeability of lipid membranes. There is a general consensus that this phenomenon does not depend on the fine chemical structure of the diffusant and membrane, but rather depends on the collective properties of the whole membrane. Density (or volume) fluctuations are the likely cause of the temperature-dependent permeability of lipid membranes. Interestingly, the maximum permeability occurs near the main phase transition of lipid monolayers[214] and bilayers,[215] where the amplitude of the density fluctuations reach a maximum (see sections *Static and Dynamic Volume compressibility* and *Area compressibility*).

Papahadjopoulos *et al*[216] were the first to demonstrate that the permeability for sodium ions (they used radiolabeled^22^ Na^+^ ions) increased by at least a factor of 100 in the phase transitions of dipalmitoyl phosphatidylglycerol (DPPG) and dipalmitoyl phosphatidylcholine (DMPC), in agreement with the phase transitions of these lipids, as measured by the fluorescence changes of embedded markers. The permeation profile for DPPC was found to be similar. It was demonstrated that cholesterol both abolishes the permeability maximum and the chain melting discontinuity. Along the same lines Mouritsen *et al*[217] and Sabra *et al*.[218] found that the permeability of dimyristoylphophatidylcholine (DMPC) membranes for Co^2+^ was drastically enhanced in the phase transition regime. These authors also demonstrated that the insecticide, lindane, changes the permeability. Jansen and collaborators[219] showed that membranes in their transition are much more permeable to water. In a recent series of articles, Heimburg and his coworkers had shown that the passive permeability P is strongly related to the area compressibility defined by eq. (18) through the relationship:[220]

(32) $$P=P_{{}^{\circ}}+const\;\cdot \;{K_{T}}^{A}$$

where *P_o_* is the ideal permeability in the absence of fluctuations. As compressibility changes are proportional to specific heat variations, ∆*c_P_*, obtained by DSC measurements, eq. (31) can be re-written as

(33) $$P=P_{{}^{\circ}}+const'\;\cdot \;\Delta c_{p}$$

The validity of eq. (33) has been experimentally tested. Analogous arguments can be set forward for what concerns the electrical conductivity.[221,222] Finally, pore formation in membranes by the inclusion of antibiotic peptides[223,224] has also been studied by ITC.

### Membrane stability and solubilization

Membrane stability can be directly quantified in terms of the free energy of the mixed membrane compared to the free energy of the most favorable alternative structure. For micelle-forming additives, the free energy of the alternative micellar state can be approximated by that of pure additive micelles, as the freedom of micelles to vary their size and shape renders mixing in micelles typically close to the ideal. Let *CMC* be the critical micellar concentration and *K_o_* the partition coefficient, then, the standard chemical potential difference of the solute between bilayers and micelles will be,

(34) $$\Delta {{µ}_{S}}^{0,\;b\to m}=RTln\left(K_{{}^{\circ}}\cdot CMC\right)$$

Which can be considered as an indicator for membrane destabilization by micelle-forming solutes.[135] Molecules perturbing the membrane, already at a low concentration, show $∆{µ}_{S}^{0,b\to m}$ < 0, that is, *K_o_· CMC*<1. Molecules with *K_o_· CMC*<1 do not destabilize the membrane at low concentration, but may solubilize membranes due to cooperative effects at very high additive concentrations.

Another approach to shed light on the membrane-disordering effects of additives is to investigate their effect on the melting temperature *T_m_* and other characteristics of the gel-to-liquid crystalline transition of a model lipid. As discussed in section *Modification of the membrane phase diagram by solutes*, an additive that disorders the membrane can be expected to favor the fluid phase over the gel phase so that the *T_m_* is lowered. ITC is an excellent method to study membrane solubilization, which is thought to be a surfactant-induced, lamella-to-micelle transition.[225] Similarly, ITC can be also be successfully employed to study the reconstitution of vesicles on addition of lipids to a micellar lipid-surfactant system.[132,142] This method does not detect the lamellar or micellar state *per se*, but the trend of the system to form micelles or vesicles. Below the critical concentration for solubilization, the injected surfactant micelles dissolve, and the surfactant is partially inserted into the membrane depending on the membrane-water partition coefficient of the surfactant and on the surfactant-to-lipid ratio.[139,193,226] This micelle-to-membrane transfer is typically endothermic. The appearance of the first stable mixed micelles in the system cannot be detected by structural methods, as virtually all the material is still in a lamellar phase. However, it reverses the direction of the surfactant transfer, injected surfactant micelles persist now and extract surfactant (exothermic) and lipid from the vesicles. This leads to a sudden jump (usually accompanied by a reversal in sign) of the heat of titration. The surfactant-induced lamella-to-micelle transition of lipid systems has also been studied by DSC.[227] The transition of fluid lipid bilayers to the inverse hexagonal phase can be induced by increasing temperature (monitored by DSC) or by the addition of compounds or changes in ionization, inducing negative spontaneous curvature.[228] For a recent review of the broad field of the membrane-surfactant interactions see, for example, Keller *et al*.,[229] Garidel *et al*.[230] and Heerklotz.[231]

### Membrane fusion

Some attempts have been made to employ calorimetric techniques to investigate the fusion events among lipid vesicles. The time evolution of the DSC thermograms of a suspension of lipid vesicles is a clear indication of the occurrence of fusion events among the particles. Such an observation has been exploited in different ways. Consider, for instance two suspensions of vesicles of different lipid composition rapidly mixed at time *t* = 0. The bilayer of the two vesicles undergoes a different melting temperature, therefore, if they appreciably differ, the associated DSC thermograms show two distinct, well-separated peaks. After mixing we observe a gradual shift in the temperature transition proportional to the amount of fused vesicles. The basic requirement for applying this technique is that the fusion rate must be very slow in comparison to the time-scale of a typical DSC run.

Another interesting application of DSC is the relationship between the fusion rate of the lipid vesicles and the physical state of the vesicles’ lipid bilayer. It has been generally observed that the fusion rate (determined by fluorimetric techniques) rapidly increases above the gel-to-liquid crystalline phase transition (determined by DSC).[232,233]

The fusion of viruses with lipid vesicles has been studied using ITC.[162,234] As the integral heats of titration do not provide any information on transient states, the heat of fusion of the bilayers *per se* is small, and the heat effects observed should be mainly attributed to interactions of viral proteins with the target membrane. For example, a partial deprotonation of a viral protein on membrane fusion was detected by ITC using the buffer variation method (see section *Isothermal titration calorimetry*).[162] Also the enthalpy of proton-induced vesicle fusion was measured by ITC.[228] DSC studies of viral proteins have yielded important information on fusogenic protein states in viruses (see the next section).

Finally, the measurement of another thermodynamic parameter, the volume compressibility (performed by acoustic techniques as described in section *Static and Dynamic Volume compressibility*), has been applied to investigate the well-known phenomenon of polymer-enhanced fusion of lipid vesicles, by exploiting the relationship between compressibility and vesicle surface hydration.[235]

## Stability and Partitioning of Proteins in a Lipid Environment

The fundamental issue of the insertion of a hydrophobic protein into a lipid membrane has stimulated an extremely large number of studies. On account of the complexity of the problem, several attempts to capture the main factors involving the energetics of protein insertion into the lipid core have been done. We list a typical thermodynamic description of the whole process:

### Hydrophobic effect

The free energy gained from the hydrophobic effect on the incorporation of proteins into a lipid bilayer can be calculated in two ways: (i) On the basis of the amino acid sequence and the free energies of transfer of individual amino acid side chains from water into the vapor phase[236] or into hydrocarbons,[237] for a recent critical analysis of the transfer energy of a protein into different solvents see, e.g., the study of Simon *et al*;[238] or (ii) from the change of the protein / water interfacial area together with a value for the free energy change per unit area.[239] From studies of hydrocarbons and hydrophobic amino acids, the free energy per area was found to be 20 – 25 cal/(*mol* · A^2^), while the interfacial area is that area of a protein molecule that is accessible to water molecules. It can be estimated by describing the protein as a sphere or cylinder or by numerically evaluating the true surface area by standard packages, which calculate the solvent inaccessible regions for any protein geometry.[240] This energy strongly depends on whether the protein is in a helical or unfolded conformation. As the hydrophobic effect originates in the reduction of the mobility of water molecules, it is predominantly of an entropic nature. The enthalpy change is relatively small, less than a few kcal/mol, and it is often neglected.

### Hydrogen bonds and conformational changes

If hydrogen bonds between protein and water molecules are broken by the incorporation of the protein and not restored in the membrane, an energy of about 5.8 kcal/mol of the hydrogen bond is lost.[241] To prevent this large loss of energy, protein molecules in the membrane adopt a conformation that allows the intramolecular formation of hydrogen bonds. This is optimal in an α-helical conformation. Hence, the hydrogen bonds are the cause for the frequently observed α-helical conformation of membrane-incorporated protein segments. Considering the final conformation of the protein in the membrane as an a-helical, the change in the internal degrees of freedom depends on the protein conformation in water. If the protein in the water is also helical, the internal degrees of freedom do not contribute to the free energy change. If, however, the protein in water adopts an unfolded conformation, internal degrees of freedom become lost on incorporation in the lipid matrix. The corresponding free energy change can be estimated for helix-coil transitions and amounts to 1.2 kcal/mol of the residue.[242]

### Protein immobilization effect

The change in free energy due to the immobilization of external degrees of freedom of a protein, on incorporation, can be easily estimated. The protein in water is treated as a freely moving particle; its free energy given by that of an ideal gas. In the membrane it is treated as completely immobilized without an energy cost. The change in free energy of the translational degrees of freedom can then be calculated from standard formulas of statistical thermodynamics. In the case of a bilayer-spanning protein of 20 amino acid residues with a molecular weight of about 2,000, at *T* = 300 K, one obtains ∆*G*≈ 10 kcal/mol. Under the same assumptions, immobilization of the rotational degrees of freedom yield approximately the same value, thus, protein immobilization is found to involve an energy change of about 20 kcal/mol. The above-mentioned figures can be slightly modified by allowing a partial retention of the freedom degrees of the protein within the lipid bilayer, but the overall picture remains unchanged.

### Perturbation of the lipid matrix

Protein insertion gives rise to a significant alteration of the lipid bilayer order parameter at the protein-bilayer periphery. The effect is even more dramatic if the lipid bilayer and the protein inclusion have different thicknesses. This point is very delicate and arises from a subtle interplay of different contributions, requiring the knowledge of the order parameter of the lipid chains, near the lipid-protein interface. Neglecting for a moment, the problem of different lipid-protein heights, the lipids contacting the included proteins lose several of their internal degrees of freedom just for geometrical reasons. The lipid molecules are strongly coupled with each other, this local anomalously large order parameter slowly relaxes with the distance from the lipid-protein interface. Things are even more complex if one includes lipid-protein height variations. As stated in section *Lateral phase separation: different routes to domains formation*, the different height between a membrane protein and the lipids generate a curvature of bilayer thickness around the protein: in order to minimize this unfavorable arrangement, proteins attract each other, eventually leading to the formation of large protein clusters.

The sum of the four energies described above is the clue for membrane-water partitioning and protein conformational transition. As a rule of thumb: Because the net number of hydrogen bonds is not significantly changed on going from a helix to a water-solvated coil, the aqueous helix-coil transition is approximately isoenergetic.

Water-lipid partitioning of the helix is estimated to be about 30 kcal/mol in favor of the lipid by virtue of the hydrophobic effect: exposing a hydrophobic helix to water will dramatically reduce water entropy.

The water-lipid partitioning of the coil is estimated to be about 40 kcal/mol in favor of the water, due to the loss of protein-water hydrogen bonds on entering the bilayer.

The resulting energy estimated by the above thermodynamic cycle must be augmented in the presence of interactions between the different helices belonging to the same protein and embedded into the lipid matrix.

A large number of articles dealing with the key issue of the energetics of membrane partition into a lipid bilayer can be found in the literature. A non-exhaustive list of some representative articles is as follows: Janhig,[243] Popot *et al*.,[244] Ben-Tal *et al*.,[245] White *et al*.,[246] Engelman *et al*.,[247] and Babakhani *et al*.,[248] while for some representative reviews on the simulation of lipid-protein interactions see, e.g., Biggin and Sansom[249] and Bond *et al*..[250] As stated before, membrane partitioning and helix formation are strongly coupled. On the experimental side, studies on this link are not always investigated and many studies focus on the helix stability alone.

The denaturation behavior of membrane proteins has been studied by DSC in reconstituted vesicles as well as in whole viruses or cells; for a review see Shnyrov *et al*..[251] It is worth mentioning that most membrane proteins seem to exhibit smaller enthalpies of denaturation (≈14 kJ g^-1^) than typical soluble proteins (≈33 kJ g^-1^), suggesting that the membrane stabilizes some residual structure.[252] Wieprecht *et al*.[253,254] claimed that they could separate the conformational and partitioning effects by ITC experiments, by comparing all-L peptides with DD-isomers, which should show the same hydrophobicity, but are not (or are less) capable of forming a helical structure.

## Kinetics Phenomena

### Heating and cooling modes DSC

Indirect information on the kinetics of transitions from an ordered to a disordered lipid configuration can be obtained by investigating the effect of the DSC scan rate on the apparent transition temperature and the shape of the DSC peaks.[11,255] This is a well-known general chemicophysical effect, independent of the peculiar nature of lipids and valid for any melting process of simple, point-like molecules, where molecules can be arranged in an ordered (solid) regular lattice on a disordered (fluid) structure. The extremely large number of internal degrees of freedom, typical of lipid molecules, introduce additional effects due to the coupling between positional and internal order parameters of the lipids. Let us start with the simplest picture. The process of phase transformation is studied by driving an initial phase into a region of the phase diagram where it is metastable or unstable. Hysteresis is usually observed during phase transformation. According to the two-dimensional nucleation theory,[256,257] the transformation from a disordered to an ordered phase requires the formation of a so-called critical nucleus, or gel domain when we consider lipid bilayers. The free energy ∆G to form a gel domain in the fluid phase is given by the expression

(35) $$\Delta G=\Delta µn+2\gamma \;{\left(\pi \sigma n\right)}^{1/2}$$

where ∆*µ* denotes the temperature-dependent chemical potential of a lipid in the gel with respect to the fluid phase, g the line tension between the fluid and gel phase,[258] n the amount of lipid constituting the nucleus, and s the area per lipid in the gel phase. Above the main phase transition temperature, both terms are positive and only small nuclei can form (so-called heterophase fluctuations).[63,259] Below the transition temperature, the chemical potential in the gel phase becomes lower than that of a lipid in the fluid phase, driving the transformation. However, this driving force is opposed by the line tension arising from the gel-fluid interface. There exists a critical nucleus size n*=*πσγ^2^*/(∆*µ*)^2^ for which the free energy exhibits a 0maximum ∆G*

(36) $$\Delta G^{*}=\frac{\pi \sigma \gamma^{2}}{\Delta µ}$$

Gel nuclei with a size n < n* are unstable and will dissipate. Nuclei with n > n*, however, will grow, thereby transforming the entire system into a state of lower free energy, that is, the gel. The time t* required to overcome this barrier will scale as t*≈exp(∆G*/*kT*). For a system quenched to a temperature much lower than the transition temperature, ∆*µ* becomes large and ∆G* vanishingly small. In this case, there is almost no impediment to the phase transformation process. On the other hand, at a temperature close to the phase transition temperature, both the critical cluster size and the time required to form the critical cluster diverge. If cluster growth results from the (reversible) addition of single lipids to the cluster boundary, the speed of gel phase propagation is given by:[63]

(37) $$u=u_{MAX}\;\left(1-\exp\left(\Delta µ/kT\right)\right)$$

where u_MAX_ denotes the maximum achievable speed when the probability of the reverse process can be neglected. Therefore, if the cooling rate of a typical DSC experiment is fast, only a limited number of solid nuclei begins to form and grow inside the membrane, which remains in a fluid undercooled state. This has a deep influence on the position intensity and shape of the calorimetric peak detected by the DSC measurement. Direct experimental evidence for the nucleation and growth mechanism in lipid bilayers is difficult to obtain. Within the framework of heterophase fluctuations, Kharakoz *et al*,[63] were able to derive a kinetic model explaining the ultrasonic anomalies observed in experiments on multilamellar vesicles. By fitting to the kinetic model, estimates of the line tension and the thermodynamic driving force can be obtained. Direct visualization of the initial stages of cluster nucleation and growth has thus far only been achieved for two-dimensional colloidal systems, by using fluorescent probes more soluble in the fluid phase. This picture has been recently confirmed by detailed Molecular Dynamics simulation.[260] Very good agreement with the classical two-dimensional nucleation theory has recently been reported for colloidal nucleation driven by an electric field, which allows precise control over the thermodynamic driving force.[261]

The different transition temperature values observed in the heating and cooling modes and described earlier, is a reversible phenomenon observed in most of the lipid systems. A limited number of lipid systems show irreversible effects observed only in the first temperature run. Sometimes people observe a temperature shift, or even the appearance of a peak, just in the first DSC run; following that the peak remains constant in all the subsequent scans. A typical example is given by glycolipids, and especially from a glycolipid subclass: the gangliosides. In these lipids the head is bulky (5 – 7 sugar units) and has a size comparable to that of the tails. Tightly packed head groups may show cooperative effects similar to those observed for the tails. Therefore, the system exhibits a richer phase behavior as extensively studied by Corti and coworkers by DSC and structural techniques.[262,263]

### Kinetics of phase transitions

In the previous section we have investigated non-equilibrium phenomena in lipid bilayers through DSC measurements performed at different scan rates. A complete and more direct approach, made possible by the progress in calorimetric instrumentation, exploits the response of the system to a sudden perturbation. Detailed studies of the kinetics of lipid phase transitions, in the absence and presence of additives, have been performed by measuring the time-dependent thermal response of lipid samples to periodic pressure modulations[32] and pressure jumps.[27,35] Experimental results evidence a good relationship between the temperature-dependent relaxation times of chain melting and heat capacity. Small amounts (1 mol%) of cholesterol added to DPPC reduce the relaxation time, τ, by a factor of 4,[27] whereas, about 1 mol% of the anesthetic dibucaine increases τ two-fold.[31] Often these effects have been interpreted as being related to the size of the cooperatively melting clusters in the membranes. In a recent series of articles, however, Heimburg and his coworkers suggested a different interpretation. Starting from the theory of thermodynamic fluctuations and the Landau ansatz for the relaxation rate of out-of-equilibrium phenomena, Heimburg *et al*. derived a linear relationship between the relaxation time τ and the specific heat *c_P_*.[27,264] They used the standard Landau assumption that a relaxation of any ‘order parameter’ *S* is proportional to its distance from the equilibrium value *S_eq_*:

(38) $$\frac{\partial S}{\partial t}=\;-\Lambda \frac{\partial G\left(S-S_{eq}\right)}{\partial S}$$

Where *G(S – S_eq_)* is the free energy of the system expressed as a function of the deviation from the equilibrium *S – S_eq_* and Λ is a viscosity-related mobility factor. Thus –∂G(S –S_eq_)/∂S plays the role of a thermodynamic force, driving the system to a new equilibrium in response to an external perturbation. A calculation gives an exponential time decay: *S –S_eq_* ≈ exp(–*t/τ*), where

(39) $$\tau \approx \frac{RT^{3}}{L}c_{p}$$

L being a phenomenological constant. The linear relationship between τ and *c_P_* foreseen by eq. (39) has been indeed experimentally tested.

Some studies have also been performed on the kinetics of the transition from the ordered gel phase to the undulated (ripple) phase in phospholipid vesicles.[49]

### Kinetics of solute sorption and exchange

Isothermal titration calorimetry provides information on the kinetics of re-equilibration after injections.[265] Membrane partitioning of solutes is often fast compared to the time constant of fast calorimeters (≈ 15 s). If the penetration of the solute to the inner monolayer occurs within a few minutes, the heat peaks may exhibit a biphasic behavior.[137] Water sorption calorimetry with small sudden changes in RH (the relative humidity) reveals the swelling to occur within about ten minutes if the film is thin enough and the gas flow is sufficiently fast; an interpretation in terms of system kinetics is hardly possible.

Even if kinetic constants are not of particular interest, it must be guaranteed for a thermodynamic evaluation of the data that the system reaches an equilibrium during the experiment. For example, the next injection of an ITC run should only be made after a sufficient time for the heat response to reach the baseline level. It must, however, be stressed that this is necessary, but not a sufficient criterion for having reached the equilibrium, as the re-equilibration of the system after an injection may exhibit complex kinetics involving processes with different time scales. In most cases, there is a simple, but very effective means, to rule out problems arising from slow processes: to combine up- and down-scans or scans with different speeds. This is a routine in the DSC of lipids. In ITC, it is advisable to combine, for example, the uptake and release or solubilization and reconstitution experiments, to rule out incomplete equilibration. In sorption calorimetry, it is useful to compare up- and down-scans in RH. PPC performs up- and down-jumps in pressure routinely, thus allowing one to recognize the irreversible effects and metastable states occurring in a transition.[266]

In a recent series of articles[267–269] we investigated by DSC, the transient variation of the calorimetric peak associated with the lipid main transition of multilamellar one-component vesicles, incubated at different times with a diffusant impurity dissolved / dispersed in the buffer solution. In the early stages of the sorption process the DSC scan showed a single narrow calorimetric peak, typical of a pure lipid bilayer. At longer incubation times the peak broadened and shifted in temperature, and finally, on approaching the equilibrium distribution of the impurity between the lipid and water the peak became narrow again, but the transition temperature shifted to a new position. This effect was due to the unequal distribution of the drug between the outer and inner bilayers of the multilamellar vesicles during the partition / permeation kinetics.

As discussed in section *Modification of the membrane phase diagram by solutes*, impurities shift the transition temperature of a bilayer in a way dependent on their local concentration. What we observe at intermediate times is just the convolution of signals coming from regions with different concentrations of the impurity. At equilibrium the two-peak structure merges into a unique peak because the impurity is evenly distributed over the entire multi-lamellar structure of the liposome. This finding may provide useful information about the lipid bilayer permeability and partition coefficient in model membranes. These parameters could be quantitatively measured in a series of DSC measurements performed at different times, provided a proper diffusion / partition interpretative model is developed. The obvious limitation of this technique is that it applies to slow the permeation kinetics. Studies are in progress in this field.
